# Confronting potential food industry ‘front groups’: case study of the international food information Council’s nutrition communications using the UCSF food industry documents archive

**DOI:** 10.1186/s12992-022-00806-8

**Published:** 2022-02-12

**Authors:** Sarah Steele, Lejla Sarcevic, Gary Ruskin, David Stuckler

**Affiliations:** 1 Cambridge Public Health, Forvie Site, University of Cambridge School of Clinical Medicine, Cambridge Biomedical Campus, Cambridge, UK; 2 Intellectual Forum, Jesus College, Jesus Lane, Cambridge, CB5 8BL UK; 3 U.S. Right to Know, 4096 Piedmont Ave. #963, Oakland, CA 94611 USA; 4 Bocconi University, Milan, Italy, and Intellectual Forum, Jesus College, Jesus Lane, Cambridge, CB58BL UK

**Keywords:** Industry influence, Public Health and nutrition communication, Commercial determinants of Health, Vested interests

## Abstract

**Abstract:**

**Background:**

There are growing concerns that the public’s trust in science is eroding, including concerns that vested interests are corrupting what we know about our food. We know the food industry funds third-party ‘front groups’ to advance its positions and profits. Here we ask whether this is the case with International Food Information Council (IFIC) and its associated Foundation, exploring its motivations and the potential for industry influence on communications around nutritional science.

**Method:**

We systematically searched the University of California San Francisco’s Food Industry Documents Archive, for all documents pertaining to IFIC, which were then thematically evaluated against a science-communication influence model.

**Results:**

We identified 75 documents which evidence that prominent individuals with long careers in the food industry view IFIC as designed to: 1) advance industry public relations goals; 2) amplify the messages of industry-funded research organizations; and 3) place industry approved experts before the press and media, in ways that conceal industry input. We observed that there were in some cases efforts made to conceal and dilute industry links associated with IFIC from the public’s view.

**Discussion:**

Instances suggesting IFIC communicates content produced by industry, and other industry-funded organisations like ILSI, give rise to concerns about vested interests going undetected in its outputs. IFIC’s deployment to take on so-called “hard-hitting issues” for industry, summating evidence, while countering evidence that industry opposes, give rise to concerns about IFIC’s purported neutrality. IFIC’s role in coordinating and placing industry allies in online and traditional press outlets, to overcome industry’s global scientific, legislative, regulatory and public relations challenges, leads also to concerns about it thwarting effective public health and safety measures.

**Conclusions:**

IFIC’s promotion of evidence for the food industry should be interpreted as marketing strategy for those funders. Effective science communication may be obfuscated by undeclared conflicts of interests.

## Introduction

There are growing concerns that the public’s trust in science is eroding [1, 2]. This is particularly worrisome in the midst of pandemic COVID, when rapid responses to emerging scientific information can have a profound impact on the well-being of entire societies. The reasons are multiple, including populist politicians who repeatedly blame ‘fake news’ for misinformation, as well as a decline in scientific literacy and numeracy skills amongst the general public [3]. In some cases, misinformation may occur from benign errors or arise from a general lack of scientific consensus. In others, the spread of misinformation can be deliberate, sometimes referred to as ‘disinformation’, such as when powerful groups seek to cast doubt on scientific evidence, or promote a pseudo-scientific view, in order to further their profit.

What is particularly challenging about disinformation is that those who spread it often seek to conceal their involvement, taking a lead from the past activities of the tobacco industry. This industry used various groups at arm’s length to confuse the science in the public mind about the health impacts of tobacco, so as to thwart regulation and continue to sell products, over many decades [4]. Indeed, previous studies on the commercial determinants of health have revealed how vested interests may attempt to cloak their role in various domains of public health communications [5, 6]. One analysis of e-cigarette tweets discovered that industry-funded bots which appeared to be ordinary persons were being used to echo and amplify false evidence that opposed regulation [7]. Another study of email exchanges between founders of the Global Energy Balance Network (GEBN) and The Coca-Cola Company (TCCC) found that the company sought to hide its funding from public view [8]. In both of these cases, industry activity was discovered through comparisons made to existing industry documents.

Here we propose to extend on these approaches to evaluate systematically the potential for such veiled interests behind nutritional information, by using systematic searches of industry document archives to go ‘behind the scenes’ of corporate-funded scientific groups. Previous studies have used this method to explore the activities of industry-funded third-party organisations like GEBN and the International Life Sciences Institute (ILSI) and its constituent organisations around the world, revealing their funding, industry influences, and past interactions [9–12]. Indeed, here we explore one case study within the wider system of corporate efforts to influence health and science communications. For our case-study, we evaluate the International Food Information Council (IFIC) and its associated Foundation, a leading communicator of evidence in public health and nutrition. IFIC comprises two legal entities, including a US-based 501(c)(6) trade association and an educational arm it created in 1991 called the IFIC Foundation, a US-based 501(c)(3) organization [13]. IFIC’s main website, foodinsights.org, reports that, consistent with its constitution as a non-profit body, it seeks to “effectively communicate science-based information on health, nutrition and food safety for the public good” [14]. It notes that IFIC does not speak for or represent ‘any company, industry, product or brand’, and it asserts that it brings together, and works with, health and nutrition professionals, educators, government officials, and food, beverage and agricultural industry professionals [14]. However, its funding sources are multiple, but are not widely and well-disclosed, with draft Internal Revenue Service documents showing that it has previously received contributions from PepsiCo, Mars Inc., Kraft, Monsanto, and TCCC, among other food and agricultural entities [15, 16].

IFIC’s communications have long been a source of controversy. Recently, Bellatti and colleagues have decried IFIC and its associated its Foundation, as a “front group”, in view of how it has voiced strongly against the role of sugar and sugar-sweetened beverages in obesity epidemics [13]. More generally, they suggest that IFIC promotes a skewed portrayal of evidence, disseminating only research which is favourable to industry. They argue uses IFIC’s seeming credibility can reach the press, policy makers, and the public at large in which its underlying funders cannot due to their over competing interests [13].

If IFIC and its Foundation are, in fact, non-profit, neutral scientific bodies, how would we know? In any scientific study, it is necessary to test and falsify hypotheses. To show that IFIC are not what they claim to be would require an attempt at falsification. Should there exist even a few cases of clear industry influence, it would negate IFIC’s claims. This is analogous to how efforts to disprove that ‘all swans are white’ requires evidence of but one instance of a black swan. While it is possible that IFIC promotes a great deal of credible evidence, this could relate to areas which have little or no impact on their funders but is used to build its scientific reputation for when it does matter to their funders.

Thus, here we perform a systematic search of documents in University of California San Francisco’s Food Industry Document Archive (UCSF FIDA), available at https://www.industrydocuments.ucsf.edu/food/. We threaded this evidence against an adapted version of Sacks and colleagues’ ‘science-communications influence’ framework, covering three main arcs of intention to influence: 1) evidence generation or summation; 2) pressure on bodies and decision-makers; and 3) cultivating relationships with policymakers, professionals and the press to influence the public [5]. Of note, for our study, we cannot test or demonstrate actual influence, but only intent to influence, and we aim to test IFIC’s assertion that it does not represent its corporate funders. Such an exploration is critical as IFIC is merely one entity in a larger system of corporate-funded scientific communications co-option, which past studies have suggested may be coordinated around the world, and used to assert corporate interests over public health [10].

## Materials and methods

Our search was based on the UCSF Industry Documents Archive, a digital archive of industry documents, that since 2002 has brought together documents on a range of industries to facilitate open access to industry materials, and to facilitate research on the commercial determinants of public health. More specifically, the Food Industry Documents Archive contains those documents pertaining to the food industry’s activities, including advertising, marketing, regulatory activities, and scientific research. At the time of search in October 2019, the entire UCSF database comprised 92,050,662 pages of 14,971,530 documents, of which 391,373 pages in 90,823 documents were relevant to food industries [17].

**Fig. 1:**
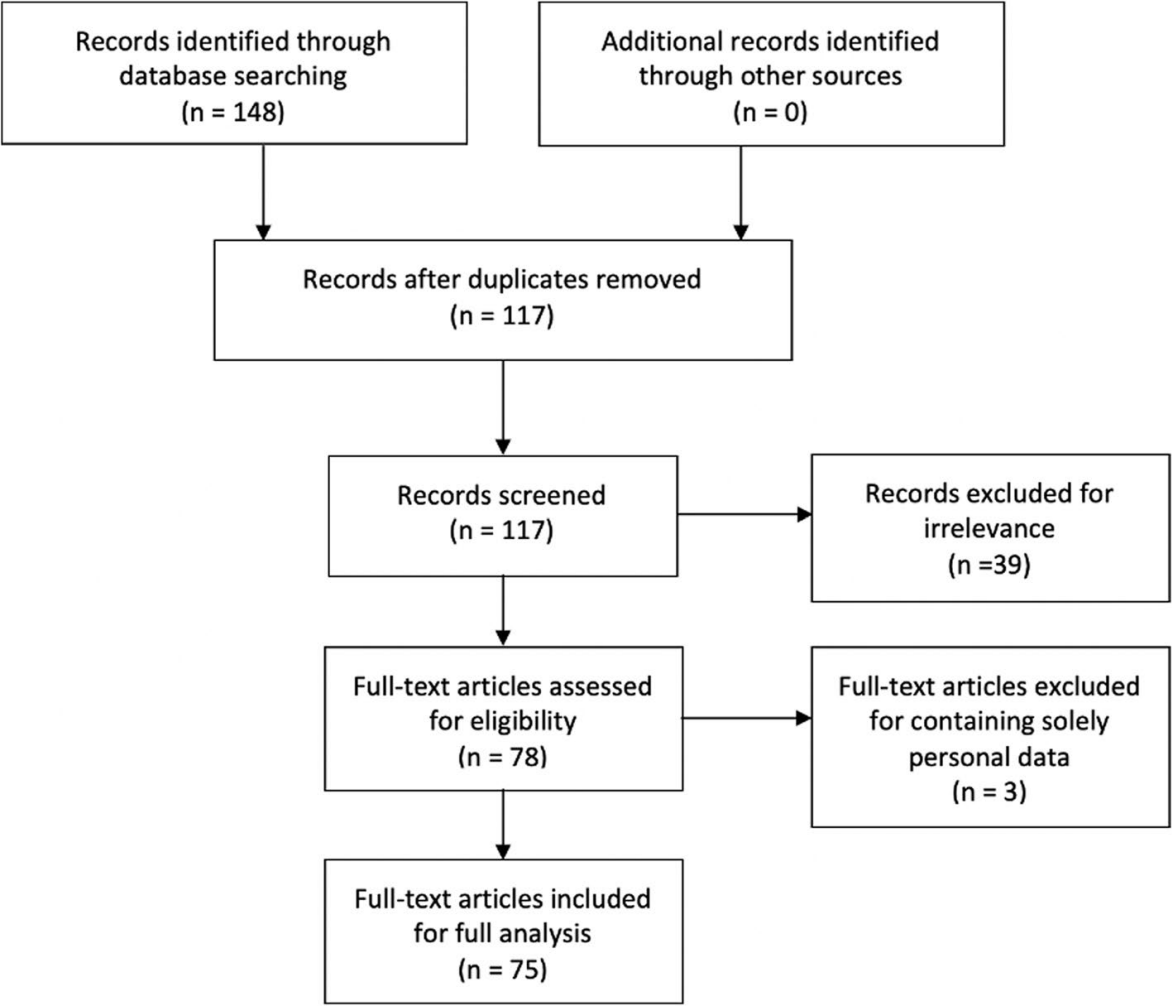
Flow diagram

Two researchers (SS and LS) searched the database using key terms “IFIC” or the “International Food Information Council”, as these capture discussion of both the organization and its Foundation. Figure 1 summarises the study inclusion/exclusion parameters. The search identified 148 documents on IFIC, of which 31 were duplicates. The remaining set was then screened for further inclusion. Communications were excluded if they did not discuss IFIC’s role in working with researchers, government, industry, trade organisations, policy bodies and other relevant organisations or individuals to shape messaging for public consumption as relevant and worthy of further investigation for the purposes of this research (*n* = 39). Documents were further excluded if the communication was solely personal in nature (e.g., arranging clearly personal dinners or events, or talking about illness and bereavement) (n = 3). Taken together this yielded 75 documents in the final analytical document set.

The analytical set of 75 documents were then thematically mapped and evaluated against the science-communication influence model, on three domains: (1) influence on evidence generation and summation; (2) influence over bodies and associations; and (3) the cultivation of relationships with policymakers and opinion leaders [5]. This model was adapted from Sacks and colleagues 2018 study of industry influence, which identified these types of activity [5]. Two researchers read the documents and mapped these activities and domains of influence. To do so, the researchers close read the documents for instances that either confirmed or refuted conduct in alignment with these, extracting quotes for further analysis and comparison to prior students in the field like those conducted by Greenhalgh [18]. We sought, where possible, to triangulate the documents against other studies’ findings in order to contextualise and validate the observations in the emails and documents. The full research team then compared and discussed the findings and analysed the documents for instances where its role, activities or funding were discussed.

Of note, we sought to distinguish IFIC and its associated Foundation, but rapidly realized this was not possible, as IFIC was referred to monolithically as a sole entity, which reflects its clearly entwined constitution and nature. Unfortunately, none of the documents clearly specified or distinguished IFIC from its Foundation. However, if we were unable to ascertain the difference, neither could the public or other recipients of their communications.

## Results

We sequentially present the three main domains of potential influence:

### Domain 1: evidence generation or summation

We found evidence that IFIC seeks to disseminate research, knowledge, or guidance, widely and to diverse audiences, in alignment with the objectives of both IFIC and its Foundation [19, 20]. However, in 41% of the documents (*n* = 31) this process of summation appeared to be exposed to attempted influenced by industry leaders and in ways that would be favourable to industry.

To give a few examples, in one email Alex Malaspina, a former senior vice president at TCCC, forwards an email from Michael Ernest Knowles, a former TCCC vice president of global scientific and regulatory affairs, and former president of ILSI, to Clyde Tuggle, former Senior Vice President and Chief Public Affairs and Communications Officer at TCCC, condemning a study on artificial sweeteners, which were being attacked widely in the press for negative impacts on health. Malaspina notes IFIC’s role in supporting industry to counter the unfavourable press, stating:

*Dear Clyde: Every one (sic) I asked including some top experts in the field believe that the Israeli study is full of holes, is not reproducible. And no respectable scientist considers it valid. In summery,(sic) it has nothing to do with human consumption of non-caloric sweeteners. How it got so much publicity is an indication of the extensive animosity that exists against our industry, which, as I have been advocating to Don, we must seriously attempt to change. The IFIC document is excellent.*

This email suggests that IFIC’s summation of evidence may have been used to counter or respond to scientific communications which were perceived as unfavourable to industry positions.

It is clear that some industry partners believed IFIC could play a role in producing ‘friendly’ evidence. Here is one example of an exchange between Malaspina and Herve Nordmann, Director Regulatory & Scientific Affairs at Ajinomoto Inc., reveals how industry approaches IFIC directly to undertake such a role:

*Dear Herve: By all means you can mention my strong endorsement to ISA and IFIC EUROPE. I will check with my Coke Friends if they can ask IFIC or the Calorie Control Council to undertake the task of translating and publishing it. Maybe you can give me a call at home…* [21]*.*

In another example, IFIC was involved in efforts to respond to concerns about the health risks of artificial sweeteners is set out in other emails:

*Dear Alex, First of all, thank you for your suggestion to contact IFIC/EUFIC [European Food Information Council]. If my memory is good, they both have been one more of your good initiatives when aspartame first become under attack from activists and consequently the press. …* [22]*.*

These exchanges, which have ific.org email addresses frequently in the recipient lists, notably make clear the science communications function of IFIC is viewed by influential persons and past and present industry executives as part of public relations and product defence efforts around sweeteners.

We also find that IFIC does this by convening a relevant network of experts. This is expressed in one email about media conference call from 2015:

*This morning we had 40+ journalists participate in our DGAC [Dietary Guidelines Advisory Committee] report conference call (see resulting media coverage and bulleted overview below). Participants included the Associated Press, Politico, WBEZ-Chicago, Capitol Press, and trade press as well as nutrition columnists and bloggers. The former DGAC panelists included Dr. Cheryl Achterberg, Dr. Joanne Lupton, Dr. Linda Van Horn, Dr. Theresa Nicklas, Dr. Connie Weaver and Dr. Roger Clemens. This hour-long webcast was recorded and has been posted to our website. We also live tweeted and heavily promoted our new Dietary Guidelines Infographic during the call. Today's combined Dietary Guidelines communication activities have resulted in more than 393,500 total impressions. In addition to the media call, we have compiled a list of 20+ experts with content-specific expertise in DGAC "hot-button" issues (Added Sugars, Aspartame, Caffeine, Sustainability, Cholesterol, Red vs. Lean meat, etc.) who have agreed to be available for media inquiries* [23].*.*

In another email chain, Malaspina states:

*I think that IFIC should follow with further media calls on the key issues raised with again former panel members but reinforced with recognized experts in the subject of the call - for us it would be a call on aspartame to rebut the aspartame allegations* [24]*.*

What we see is that, to summate and disseminate information, IFIC appears to play a role in convening networks of persons with scientific or nutrition expertise who it views as espouse positions favourable to industry or product defence, and to place these persons into relevant communications endeavours when needed.

### Domain 2: influence over public health bodies and organizations

Just over half of the documents (52%, *n* = 39) detailed IFIC collaborations with other bodies and organizations. As noted in several cases, although there was evidence that IFIC partnered with prominent public health organizations, these could not be established as favourable or unfavourable to industry positions. For example, IFIC worked with the National Institutes of Health to co-author and co-sponsor public diet and health advisory pamphlets, including “A Teenager’s Guide to Better Health.” [25] It also was included on one study from the Mayo Clinic titles ‘Moderate Cardiorespiratory Fitness is Positively Associated With Resting Metabolic Rate in Young Adults.’ [26]

However, the documents reveal that industry leaders viewed IFIC as playing a strategic role in media communications on science, as expressed in another email from Malaspina to John C Peters, a researcher at the University of Colorado, in which Malaspina summarizes the purpose of IFIC as follows:

*… IFIC is kind of a sister entity to ILSI. ILSI generates the scientific facts and IFIC communicates them to the media and public…* [27]*.*

Consistent with this view, we observed a frequent pattern of coupling of IFIC with ILSI. In on set, David B. Schmidt, then IFIC and IFIC Foundation president and CEO, emails several recipients recognizing that not only do ILSI and IFIC share the same founding industry leader, Dr. Alex Malaspina, referring to ‘his vision’, as follows:

*Dr. Malaspina made possible in founding both ILSI and IFIC and how his vision is being carried out today* [28].

These email chains reveal that IFIC’s formation and conduct is intended to be grounded in joint endeavour with other industry allies including ILSI. One such instance occurred in a set of meeting minutes from 1990, which suggest that ILSI should:

*…[l]iaise with IFIC regarding the development of a manual on risk communication for the food industry [similar to the manual published by the Chemical Manufacturers Association, (CMA)]. Following publication of this manual, sponsor workshops on risk communication, using the manual as a guide for industry, regulators, and possibly journalists. This would also be done in conjunction with IFIC.*

*4. Monitor the activities of the Residue Committee with regard to the release of the National Academy of Sciences (NAS) report on pesticide exposure in the diets of infants and children to ensure that sufficient and appropriate action is planned before and at the time of its release. Ensure involvement of IFIC in the communications aspects of this effort.*

These minutes indicate that ILSI and IFIC work in collaboration, although as noted we could not verify whether or not these collaborations were realized as intended since such documents were not publicly available.

There is also evidence that IFIC played a role in the now-defunct and discredited TCCC front group, the GEBN, when a network of collaborative bodies who often work together is set out and IFIC’s role in addressing proposals around obesity is detailed [6, 29]. One email between Alex Malaspina and Michael Ernest Knowles turns to how to address “issues hitting the industry”:

*As to the generation of credible, consensus science on the issues hitting the industry - obesity and causative factors, sugar, low/no calories sweetener safety - in particular we have to use external organizations in addition to any work we directly commission (and that needs to be very carefully reviewed in fight of the BMY article I); examples are : ILSI…Scientific Societies… Medical Associations… National Academies of Science…EU/Gov’t, Research…* [24]*.*

Of note, there was special attention to also include ‘external organisations’ beyond IFIC. The discussion emphasizes IFIC’s role and partnerships with ILSI:

*… The ' One ILSI' strategy currently being developed should do this but it's too slow these issues need to be addressed now in the traditional manner of ILSI -in a transparent manner with the best international experts and the full proceedings published and further publicized by IFIC…*

*We will look to establish partnerships with global organizations including, but not limited to, the American Society for Nutrition, ACSM, ECSS, The Obesity Society, ILSI, IFIC, IFT and others that would be sympathetic and supportive of our initiative and would highlight our message* [24]*.*

The emails suggest that a broad network of bodies act together in a supportive manner, including IFIC, sometimes to promote agreed messaging around “energy balance” and obesity and in product defence more generally. This supports prior research which identified a constellation of non-profit bodies that are partially or fully industry funded work together and function in partnerships, with past financial records uncovered showing significant funding from bodies across the food and beverage sectors supporting ILSI and GEBN, for example [6, 9, 18].

For example, we identified an instance where Malaspina strongly endorses the then newly formed GEBN to IFIC’s and IFIC Foundation’s then-president, David Schmidt, suggesting that they could support and promote it to members:

*I am enclosing an email I just sent to ILSI Europe so that I do not repeat myself and inform you that I am very impressed with the Global Energy Balance Network, or GEBN. This program was developed at the University of Colorado by our friends John Peters and Jim Hill. GEBN is a very serious project to combat obesity, which is such a critical problem for the food and beverage companies. By copy of this email I am asking John to provide you with more details about GEBN. I do hope that your IFIC members become more cognizant and supportive of this most exciting project. Warmest personal regards. Alex* [30].

IFIC is clearly set out in the emails as critical partner organization for GEBN. This suggests that IFIC takes a broader role in supporting other organizations from their inception to working on day-to-day matters with them once founded, as expressed in the vision for IFIC as a communications vehicle for industry and ILSI. We therefore move, considering this networking role, to understand the cultivation of relationships that translate these networks into influence.

### Domain 3: the cultivation of relationships with policymakers and opinion leaders

IFIC’s “Form 990” filings to the Internal Revenue Service for both the parent entity and the Foundation suggest communicating with “government officials” is part of its core activities [19, 20]. However, we were only able to identify one email showing direct attempts at influencing policymakers. In this email we see discussion of an IFIC media call, where former ILSI President Michael Ernest Knowles states to Malaspina that IFIC’s call:

*… hopefully will also demonstrate to governments that they must have credible scientists in their advisory committee, or else they risk being made to took [SIC] foolish* [23]*.*

Although there was no evidence in the document set of direct attempts use IFIC to influence policymakers, it is possible IFIC sought to achieve indirect influence by communicating media and evidence favourable to industry positions.

## Discussion

Our case-study of IFIC, including its associated Foundation, reveals evidence that suggests it is viewed by some former and current industry heads as holding a role in promoting and representing industry or other commercial bodies’ interests, and even as having the potential to conduct product or ingredient defence in the face of impending negative publicity. We make several important observations from the emails we identified. First, there was evidence that IFIC was intended to communicate evidence produced by other industry-funded organisations like ILSI [9–11, 31–33]. Second, IFIC was employed to take on so-called “hard-hitting issues” to industry, summating evidence, but as well as to counter evidence that industry opposed. Third, IFIC played a role in coordinating academic contacts, major scientific bodies, public institutions, and medical associations, while working with the media to place industry allies in media to overcome industry’s global scientific, legislative, regulatory, and public relations challenges. Importantly, we also observed that there are suggestions that IFIC could act in a manner that may in effect conceal or dilute industry links in public or professional content.

Our research suggests at its heart IFIC is part of a network of actors that bring together industry with non-profits that it funds, alongside favourable experts from research institutions and universities, and the press. It acts in coalitions—with those networks—to mobilise product defence messaging or other messaging favourable to industry. Many of the individuals we observed discussing IFIC have themselves complex employment histories, working inside corporations and outside in non-profits and research. Our work, like Greenhalgh’s, observes that several core people exert influence, guiding coalitions and cooperation, including rather specifically “the MIT-trained food technology specialist Alex Malaspina (1931–), was concurrently vice president of TCCC (1969–ca. 2001; ILSI president 1978–2001)”, but also that many of the scientists engaged in emails traverse the public and private spaces, taking industry funding for specific research, and regularly sitting on industry-funded non-profit bodies as seemingly independent or neutral parties, while evidence suggests they act favourably toward in industry [18].

Such convening of experts to further industry agendas mirrors observations made by Greenhalgh around ILSI, which as we saw, is considered a sister body to IFIC. Greenhalgh observes in her work that ILSI undertook a process that led to instances where five soda and food giants engaged the services of company-friendly scientists to promote a friendly public agenda [18]. In her work using the University of California, San Francisco, Food Industry Documents Archive she shows both that 1) the focus on promoting physical activity among children was borne out of political necessity, as “… ILSI’s board argued that collective action was necessary to defuse the criticism of the industry that was building, and to forestall regulation and legislation (such as soda taxes)…”, and 2) crucially involved co-option of experts. She showed that ILSI’s project of advancing industry-friendly responses involved the assembling of “a network of loyal obesity specialists”. Many of these experts were scientists who worked both for, or with, industry, while holding public institutional status. She observes that “[b]y intervening at this early stage in the policy process to place industry-friendly researchers in important seats at the science and policy table, the soda industry was ensuring that its interests would continue to be honored at later stages” [18]. We note that many of the scientists she identified as working with ILSI to further industry interests are those active in the discussions we identify with IFIC also. As such, it is critical to look at not just the co-option of experts but the network of influence that is observable in our document set. Network analysis of IFIC’s relationships is therefore important for future research.

`It is also notable that we identified direct connections between GEBN and ILSI with IFIC., Greenhalgh’s recent work, which adds context to industry endorsement and promotion of “energy balance” frameworks, details “how physical activity became the priority solution” to address emerging concerns about the obesity epidemic, and how only small industry concessions about dietary intake featured in many approaches pursued across the 1990s and beyond. Greenhalgh observes a network of organisations acting with TCCC to devise and disseminate ‘a “science of energy balance” to buttress the case for the physical activity solution to obesity’. [18] She details the critical role ILSI, then GEBN, played in promoting the notion that obesity was a product of energy out not being sufficient and thereby physical activity solutions being corporate preferred public and policy interventions [18]. She notes the creation and workings of ILSI bodies and GEBN, and discusses their working with scientists and industry players, to undertake this longer term strategy of promoting “energy balance”, supporting our conclusions above that these organisations partner together. In short, her observations correlate with ours, and triangulate our observations.

Greenhalgh also helpfully details how networks of bodies and experts are deployed to impact at “the early stage in the policy process to place industry-friendly researchers in important seats at the science and policy table” [18]. She suggests that ILSI and GEBN “provided forums in which Coke and its grantees could circulate the findings and translate them into concrete interventions” [18], and therefore it is important to see IFIC’s role as a “sister organisation” in this context and for researchers to explore more the activities of IFIC to understand its collaborative endeavours and what they involved. While our documentary analysis did not extend to online explorations of events and relationships, and we did not undertake content analysis of past event or media calls here, Greenhalgh’s findings give pause to suggest such research is timely. Our findings echo more general concerns about hidden industry influence via third-party front groups. There was no evidence that IFIC presented itself to the public as a trade association with a transparent charitable education wing, or any materials which openly demonstrate its industry links. Instead, its public facing operations appear to all come under the IFIC Foundation umbrella, which states that “we do not lobby or further any political, partisan or corporate interest”.

As with all analyses of documentary archives, our analysis has several important limitations. First, as noted, we could only evaluate intent to influence or a person’s view on what IFIC could and was designed to achieve, and not actual incidences of influence. Relatedly, we could not assess the quality of the evidence produced by IFIC, which would be the scope of a future study. However, to demonstrate that IFIC may represent corporate backers, such evidence was not required. Second, the UCSF document archive is a pre-selected group of documents and does not represent the entire universe of documents available on IFIC. The archive contains documents that have been obtained by litigation, uploaded following receipt of batches via freedom of information request, or from documents uncovered by leaks. The communications therefore may be only parts of extended conversations and may be partial. It is likely that those documents most concerning from a public health standpoint were more likely to appear in the document archive. However, we also observed multiple instances where IFIC’s evidence was not linked to a clear expression of a position favourable or inimical to industry. Additionally, the archive contains communications by senior food industry figures over several decades, some of whom were speaking as former senior executives and others who held industry posts at the time of sending the communication. We note that we researched the background of each person writing the relevant document identified, to verify that they remained influential in the food industry at the time of sending the communication. Third, the documents in our sample failed to separate parent entities from their other arms and therefore distinguishing whether IFIC or the IFIC Foundation was being discussed was not possible in this study, with their roles often collapsed, reflecting the shared aims, objectives, activities, offices and staff.

Despite the limitations of the evidence analysed, the email exchanges provide insight “in their own words” on senior level understandings, including from founders, and daily operations of IFIC and the IFIC Foundation. Other similar examples, including from different time periods and geographic locations, would help to confirm the extent of these activities, so further research should be conducted in the future.

Finally, it is unclear the extent to which the observations about IFIC might be relevant to other industry funded communications organisations. There are suggestions, however, that other organisations adhere to a similar institutional model. For example, established in 1995, the European Food Information Council (EUFIC) similarly purports to be a neutral body with a mission “to produce science-based content to inspire and empower healthier and more sustainable diets and lifestyles” [34]. Future research is needed to better understand how such third-party non-profit actors funded by industry act and influence the dissemination of public health evidence for policy and practice.

## Conclusions

Our findings have important implications for IFIC as pertains to its standing as a leading nutrition communications organisation. We found evidence that some industry figures view IFIC as not only able to convene experts who are favourable to industry but does so on occasion to consolidate evidence in a way that could be product defence, especially when media or press coverage or academic studies are emerging that contain unfavourable claims around ingredients. While IFIC contends that its role is merely to promote scientific information, we found more than one instance discussed in the industry documents that suggest it acts to organize information into a “consensus science” on “hard-hitting issues” which is specifically favourable to companies that IFIC’s Form 990 drafts suggest fund either IFIC or its Foundation. We also found that IFIC plays a ‘rebuttal function’ in public debates, especially being called upon to galvanise a defence of ingredients or products when negative press has occurred. Leading industry players view IFIC as a ‘sister to ILSI’, in being central to promoting industry-favourable content in defence of products facing potentially negative press, such as aspartame, with widespread use of ific.org email addresses throughout email chains. Our past research on ILSI exposed that it acted very clearly in the past on behalf of TCCC, among other food industry funders; a point ILSI has disputed [9, 10, 35]. In sum, the evidence discovered here through search of the USCF archive is more than sufficient to negate IFIC’s portrayal that it is a neutral organization.

We argue that IFIC and its Foundation’s communications should be viewed as conducting marketing and public relations for the food industry. There is a clear need for better systems and transparency around science communications activity to manage complex veiled conflicts of interest, especially those from industry-funded entities like IFIC. As a start, following good governance practices, IFIC could publicly disclose on its website the funding it receives, including contributors, amounts, and purpose/s. Until then, we believe the starting assumption should be that IFIC acts as an agent of the food industry and be restricted from partnership with international health organisations until robust evidence is available to demonstrate that such conflicts of interest have been addressed.

This case-study of IFIC adds a critical dimension to the literature on the commercial determinants of health, adding to evidence of how intermediary organizations work to obfuscate the industry role in their evidence summation and communications. Consistent with existing research on commercial determinants of health, we find that third-parties such as IFIC can be employed by underlying corporate sponsors to influence the perception and regulation of their products, as well as to muddy the water to lead to a perception that the evidence is not clear to support regulation; an activity that we know was well deployed by the tobacco industry [36, 37]. Importantly, we show how documentary evidence can be mapped into major arcs of influence systematically to reveal such attempts to influence debates. Our research also supports the broader literature emerging on why it is critical to examine the influence of corporations on public health knowledge and policy [38, 39]. Our analysis suggests the need to further research how vested interests co-opt science communications in their efforts to further their commercial interests.

## Data Availability

The analysable dataset including links to the documents identified can be found in CSV format at: https://docs.google.com/spreadsheets/d/1XDdgVasVM0euqQ8VTgdk8vvgYX-18DF4BBYpGmmZU6c/edit?usp=sharing

## Abbreviations

CMA: Chemical Manufacturers Association
DGAC: Dietary Guidelines Advisory Committee
EUFIC: European Food Information Council
IFIC: International Food Information Council
ILSI: International Life Sciences Institute
GEBN: Global Energy Balance Network
NAS: National Academy of Sciences
UCSF: University of California San Francisco
UCSF FIDA: University of California San Francisco’s Food Industry Document Archive
TCCC: The Coca-Cola Company

## References

[1] Wilson R. Polls show trust in scientific, political institutions eroding [Internet]. The Hill 2020 [cited 2020 Nov 24]. Available from: https://thehill.com/policy/healthcare/516412-polls-show-trust-in-scientific-political-institutions-eroding

[2] Kreps SE, Kriner DL (2020). Model uncertainty, political contestation, and public trust in science: evidence from the COVID-19 pandemic. Sci Adv.

[3] Roozenbeek J, Schneider CR, Dryhurst S, Kerr J, Freeman ALJ, Recchia G (2020). Susceptibility to misinformation about COVID-19 around the world. R Soc Open Sci.

[4] Apollonio DE, Bero LA (2007). The creation of industry front groups: The tobacco industry and “get government off our back”. Am J Public Health.

[5] Sacks G, Swinburn BA, Cameron AJ, Ruskin G (2018). How food companies influence evidence and opinion – straight from the horse’s mouth. Crit Public Health.

[6] Barlow P, Serôdio P, Ruskin G, McKee M, Stuckler D (2018). Science organisations and Coca-Cola’s “war” with the public health community: insights from an internal industry document. J Epidemiol Community Health.

[7] Kim AE, Hopper T, Simpson S, Nonnemaker J, Lieberman AJ, Hansen H, et al. Using twitter data to gain insights into E-cigarette marketing and locations of use: An infoveillance study. J Med Internet Res. 2015;17(11) Available from: https://pubmed.ncbi.nlm.nih.gov/26545927/. [cited 2020 Nov 24]. 10.2196/jmir.4466 PMC4642798 26545927

[8] Serôdio PM, McKee M, Stuckler D (2018). Coca-Cola – a model of transparency in research partnerships? A network analysis of Coca-Cola’s research funding (2008–2016). Public Health Nutr.

[9] Steele S, Ruskin G, Sarcevic L, McKee M, Stuckler D (2019). Are industry-funded charities promoting “advocacy-led studies” or “evidence-based science”?: a case study of the international Life Sciences Institute. Global Health.

[10] Steele S, Ruskin G, Stuckler D. Pushing partnerships: corporate influence on research and policy via the international Life Sciences Institute. Public Health Nutr. 2020:1–9. 10.1017/S1368980019005184 PMC7348693 32416734

[11] McKee M, Steele S, Stuckler D (2019). The hidden power of corporations. BMJ.

[12] Greenhalgh S (2019). Soda industry influence on obesity science and policy in China. J Public Health Policy.

[13] Bellatti A. Front groups: big Food’s behind-the-scenes strategy. HuffPost. 2014; Available from: https://www.huffingtonpost.com/andy-bellatti/front-groups-big-foods-be_b_4808997.html. [cited 2019 Jan 19].

[14] IFIC Foundation. About [Internet]. [cited 2019 Jan 18]. Available from: https://www.foodinsight.org/about

[15] Department of Treasury Internal Revenue Service. Form 990 IFIC 2013 [Internet]. USRTK. 2013 [cited 2020 Nov 24]. Available from: https://usrtk.org/wp-content/uploads/2016/09/2013-IFIC-Foundation-Draft-990.pdf

[16] Department of Treasury Internal Revenue Service. Form 990 IFIC 2011 [Internet]. USRTK. 2011 [cited 2020 Nov 24]. Available from: https://usrtk.org/wp-content/uploads/2016/09/2011-IFIC-Foundation-Draft-990.pdf

[17] UCSF library of food industry documents [Internet]. San Francisco; 2018 [cited 2019 Oct 17]. Available from: https://www.industrydocuments.ucsf.edu/food/

[18] Greenhalgh S (2021). Inside ILSI: How Coca-Cola, Working through Its Scientific Nonprofit, Created a Global Science of Exercise for Obesity and Got It Embedded in Chinese Policy (1995-2015). J Health Polit Policy Law.

[19] International Food Information Council Foundation. Form 990 [Internet]. 2016. Available from: https://foodinsight.org/wp-content/uploads/2014/06/2016-Foundation-Form-990-Public-Disclosure.pdf

[20] International Food Information Council. Form 990 [Internet]. 2017 [cited 2019 Dec 11]. Available from: https://projects.propublica.org/nonprofits/display_990/521439244/10_2018_prefixes_52-54%2F521439244_201712_990O_2018101215788995

[21] Peters JC, Malaspina A, Alex, IFIC, Council CC. [Email of John Peters about endorsement to ISA and IFIC Europe] [Internet]. USRTK Food Industry Collection; 2015. Available from: https://www.industrydocuments.ucsf.edu/docs/rjpk0228

[22] Peters JC, Nordmann H, Herve. [Email from Herve Nordmann to Michael Ernest Knowles and Alex Malaspina about the calorie control] [Internet]. USRTK Food Industry Collection; 2015. Available from: https://www.industrydocuments.ucsf.edu/docs/sjpk0228

[23] Malaspina A. Fwd: UPDTE: IFIC Foundation’s 2015 dietary guidelines media call [Internet]. USRTK Food Industry Collection; 2015. Available from: https://www.industrydocuments.ucsf.edu/docs/zycy0227

[24] Malaspina A. [Email from Alex Malaspina for update: IFIC Foundation’s 2015 dietary guideline media call] [Internet]. USRTK Food Industry Collection; 2015. Available from: https://www.industrydocuments.ucsf.edu/docs/lfbl0228

[25] WIN, Network W-CI. A teenager’s guide to better Health [Internet]. CSPI Collection; 1999. Available from: https://www.industrydocuments.ucsf.edu/docs/nzcw0229

[26] Shook RP, Hand GP, Paluch AP, Wang X, Moran R, Hebert JR, et al. Moderate cardiorespiratory fitness is positively associated with resting metabolic rate in young adults [Internet]. USRTK Food Industry Collection; 2014. Available from: https://www.industrydocuments.ucsf.edu/docs/mhpk0228 10.1016/j.mayocp.2013.12.017 24809761

[27] Malaspina A. [Email from Alex Malaspina for WHO unveils nutrient profiling] [Internet]. USRTK Food Industry Collection; 2015. Available from: https://www.industrydocuments.ucsf.edu/docs/mfbl0228

[28] Peters JC, John. [Email from John Peters to Alex Malaspina for FYI 20150125] [Internet]. USRTK Food Industry Collection; 2015. Available from: https://www.industrydocuments.ucsf.edu/docs/hkwl0228

[29] Kmietowicz Z (2015). Coca-Cola funded group set up to promote &quot;energy balance&quot; is disbanded. BMJ.

[30] Peters JC, Malaspina A, Alex. [Email from Alex Malaspina concerning ILSI interest in GEBN] [Internet]. USRTK Food Industry Collection; 2015. Available from: https://www.industrydocuments.ucsf.edu/docs/mspk0228

[31] Greenhalgh S (2019). Making China safe for coke: how Coca-Cola shaped obesity science and policy in China. BMJ.

[32] Thacker P (2017). Coca-Cola’s secret influence on medical and science journalists. BMJ.

[33] Michail N. Breaking away from bad science? Mars to leave ILSI in transparency bid. Food Navigator. 2018; Available from: https://www.foodnavigator.com/Article/2018/02/08/Breaking-away-from-bad-science-Mars-to-leave-ILSI-in-transparency-bid#. [cited 2018 Oct 19].

[34] EUFIC. Who Are We [Internet]. 2020 [cited 2020 Mar 27]. Available from: https://www.eufic.org/en/who-we-are/who-we-are-eufic/

[35] ILSI. ILSI Response to Globalization and Health [Internet]. ILSI. 2019 [cited 2019 Aug 5]. Available from: https://ilsi.org/ilsi-response-to-globalization-and-health/

[36] Mialon M, Swinburn B, Sacks G (2015). A proposed approach to systematically identify and monitor the corporate political activity of the food industry with respect to public health using publicly available information. Obes Rev.

[37] Nicogossian A. Review of the bottom line or public Health: tactics corporations use to influence Health and Health policy, and what we can do to counter them. World med heal Policy. 2010.

[38] Moodie R, Stuckler D, Monteiro C, Sheron N, Neal B, Thamarangsi T (2013). Profits and pandemics: prevention of harmful effects of tobacco, alcohol, and ultra-processed food and drink industries. Lancet.

[39] Nestle M. Food politics : how the food industry influences nutrition and health [Internet]. University of California Press; 2013 [cited 2018 Nov 15]. 510 p. Available from: https://www.ucpress.edu/book/9780520275966/food-politics

