# Correction to: Confronting potential food industry ‘front groups’: case study of the international food information Council’s nutrition communications using the UCSF food industry documents archive

**DOI:** 10.1186/s12992-022-00824-6

**Published:** 2022-03-08

**Authors:** Sarah Steele, Lejla Sarcevic, Gary Ruskin, David Stuckler

**Affiliations:** 1 Cambridge Public Health, Forvie Site, University of Cambridge School of Clinical Medicine, Cambridge Biomedical Campus, Cambridge, UK; 2 Intellectual Forum, Jesus College, Jesus Lane, Cambridge, CB5 8BL UK; 3 U.S. Right to Know, 4096 Piedmont Ave. #963, Oakland, CA 94611 USA; 4 Bocconi University, Milan, Italy, and Intellectual Forum, Jesus College, Jesus Lane, Cambridge, CB58BL UK

**Correction to: Global Health 18, 1-10 (2022)**

**https://doi.org/10.1186/s12992-022-00806-8**

Following publication of the original article [1], the authors flagged that the following statement had been erroneously missed from the Funding declaration: Progetto: Rif. 2018- 0863 “Pension reforms and spatial-temporal patterns in healthy ageing in Lombardy: quasinatural experimental analysis of linked health and pension data in comparative Italian and European perspective” finanziato da Fondazione Cariplo nell’ambito del bando “Ricerca sociale sull’invecchiamento: persone, luoghi e relazioni”.

The funding declaration has now been updated in the article.

The publisher apologizes for this omission and any inconvenience caused.
